# Classic psychedelics do not affect T cell and monocyte immune responses

**DOI:** 10.3389/fpsyt.2023.1042440

**Published:** 2023-01-20

**Authors:** Deborah Rudin, Alexander Areesanan, Matthias E. Liechti, Carsten Gründemann

**Affiliations:** ^1^Clinical Pharmacology and Toxicology, Department of Biomedicine, University Hospital Basel, Basel, Switzerland; ^2^Clinical Pharmacology and Toxicology, Department of Pharmaceutical Sciences, University of Basel, Basel, Switzerland; ^3^Translational Complementary Medicine, Department of Pharmaceutical Sciences, University of Basel, Basel, Switzerland

**Keywords:** psychedelic, LSD, psilocin, DMT, mescaline, T cell, monocyte

## Abstract

**Introduction:**

Classic psychedelics have been shown to exert therapeutic potential for the treatment of various psychiatric disorders, neuropsychiatric diseases, and neuronal damage. Besides their psychopharmacological activity, psychedelics have been reported to modulate immune functions. There has thus far been a sparse exploration of the direct immune-modulating effect of psychedelics on human immune cells *in vitro*. Since T cells are key mediators of several immune functions, inhibition of their function would increase the risk of infections.

**Methods:**

We investigated the effect of the classic psychedelics lysergic acid diethylamide (LSD), psilocin, *N,N*-dimethyltryptamine (DMT), and mescaline on the proliferation and stimulated cytokine release of primary human T lymphocytes and on the stimulated NF-κB induction of monocytes.

**Results:**

We did not observe any relevant direct immune-modulatory effects of the tested classic psychedelics in either cell line.

**Discussion:**

We concluded that LSD, psilocin, DMT, or mescaline did not directly stimulate the proliferation or cytokine secretion of primary human T lymphocytes or stimulate NF-κB induction of monocytes. Our findings support the future safe use of classic psychedelics in assisted psychotherapy in patients with life-threatening diseases where immune suppression and diminished immune function would be detrimental.

## 1. Introduction

Psychedelics are psychoactive substances that alter cognition and perception predominately through agonistic activity at various serotonin (5-hydroxytryptamine, 5-HT) receptors, whereby 5-HT_2*A*_ receptor agonism is the main psychoactive/psychedelic trigger (1–3). The so-called “classic psychedelics” comprise tryptamines, such as *N,N*-dimethyltryptamine (DMT), and psilocybin, lysergamides, such as lysergic acid diethylamide (LSD), and phenethylamines, such as mescaline. A growing body of evidence shows that psychedelics have therapeutic potential for the treatment of various psychiatric disorders such as major depression, anxiety, post-traumatic stress disorder (PTSD) as well as depression and anxiety due to terminal illnesses (4–8). Moreover, recent *in vivo* studies in rodents suggest that psychedelics may also exert positive effects on neuropsychiatric diseases and neuronal damage, such as Alzheimer’s Disease and traumatic brain injury (9, 10). Besides their psychopharmacological activity, psychedelics have been shown to be able to modulate immune functions (11, 12). It has been suggested that 5-HT_2*A*_ and 5-HT_1*A*_ receptor activation is involved in the immune-modulatory effects of psychedelics. The 5-HT_2*A*_ and 5-HT_1*A*_ receptor expression have been detected in many tissues mediating inflammatory conditions, including the brain, gut, and cardiovascular system, as well as platelets, endothelial cells, and smooth muscle cells (13–15). Thus, it has been shown that the 5-HT_2_ receptor agonist 2,5-dimethoxy-4-iodoamphetamine (DOI) suppresses tumor necrosis factor-alpha (TNF-α)-induced inflammation in rat aortic smooth muscle cells *via* a protein kinase C (PKC)-mediated pathway (16). PKC activation is a canonical pathway elicited through 5-HT_2*A*_ receptor stimulation (17).

In healthy volunteers, DMT induced time-dependent modifications in lymphocyte subpopulations after an oral application of 1.0 mg/kg body weight (18). The percentage of CD3^+^ and CD4^+^ cells decreased, whereas the percentage of natural killer cells increased, showing maximum changes after 2 h and returning to baseline levels after 24 h (18). Moreover, DMT affected the immunological phenotype in isolated mouse brain microglia. The TLR4 expression on CD11b^+^ cells and microglial NF-κβ (p65) intensity were significantly lower in cells treated with DMT (19). Similar observations have been reported for the psilocin treatment of isolated mouse brain microglia (19). In lymphocytes isolated from human blood, the 5-HT_1*A*_ receptor agonist DPAT increased cell proliferation whereas it was inhibited by the 5-HT_1*A*_ receptor antagonist WAY100.46 (20). It has furthermore been shown that 5-HT_1*A*_-mediated cell proliferation of mitogen-activated T and B lymphocytes is associated with an increased translocation of NF-κB into the nucleus (21).

There has thus far been a sparse exploration of the direct immune-modulating effect of classic psychedelics on human immune cells *in vitro*. Since T cells are key mediators of several immune functions, inhibition of their function would increase the risk of infections or even tumor formation (22, 23). Hence, besides the potential future application of classic psychedelics in the treatment of autoimmune diseases, the potential adverse effects of diminished immune function and subsequent risk for infections need to be elucidated. This is of special interest since classic psychedelics have been employed in assisted psychotherapy to treat anxiety in patients with life-threatening diseases (24, 25). Therefore, we investigated the effect of the classic psychedelics LSD, psilocin, DMT, and mescaline on the proliferation and stimulated cytokine release of primary human T lymphocytes and on the stimulated NF-κB induction of monocytes. This study aimed to further elucidate the relationship between classic psychedelics and the immune response of human T lymphocyte subpopulations and monocytes.

## 2. Materials and methods

### 2.1. Ethics approval statement

All subjects gave written informed consent for blood collection. The blood samples were obtained in an anonymized and coded form from the central blood donation of the University Hospital in Basel and the Blood Transfusion Center of the University Medical Center Freiburg. No ID number of the samples is visible so any assignment is impossible. The work, therefore, does not fall within the scope of the Swiss Human Research Act.

### 2.2. Preparation and cultivation of human immunocompetent cells

Human peripheral blood mononuclear cells (PBMCs) were isolated from the blood of adult donors. Venous blood was centrifuged on a LymphoPrep™ gradient (density: 1.077 g/cm^3^, 20 min, 500 × g, 20°C; Progen, Heidelberg, Germany). Afterward, cells were washed three times with PBS, and cell viability and concentration were determined using the trypan blue exclusion test. Cells were cultured in Roswell Park Memorial Institute (RPMI) 1640 medium (Invitrogen, ThermoScientific, Reinach, Switzerland) supplemented with 10% heat-inactivated fetal calf serum (PAA, Cölbe, Germany), 2 mM L-glutamine, 100 U/ml penicillin, and 100 U/ml streptomycin (Invitrogen) at 37°C in a humidified incubator with a 5% CO_2_/95% air atmosphere. PBMCs were additionally stimulated with anti-human CD3 (clone HIT3) and anti-human CD28 (clone 28.6) mAb (100 ng/ml; both from eBioscience, ThermoScientific, Reinach, Switzerland).

### 2.3. Cell division tracking using carboxyfluorescein diacetate succinimidyl ester

Peripheral blood mononuclear cells were harvested and washed twice in cold PBS (Invitrogen) and re-suspended in PBS at a concentration of 5 × 10^6^ cells/ml. Carboxyfluorescein diacetate succinimidyl ester (CFSE, 5 mM; Sigma-Aldrich, Merck, Buchs, Switzerland) was diluted 1/1,000 and incubated for 10 min at 37°C. The staining reaction was stopped by washing twice with the complete medium. PBMCs were activated as described above and cultured with psychedelic compounds for 72 h. Cyclosporine A (CsA, 5 μg/ml, Sandimmun™ 50 mg/ml, Novartis, Basel, Switzerland) served as a control substance for proliferation inhibition. Cell division progress was analyzed from three independent experiments with a BD FACSCalibur flow cytometer using BD CellQuest Pro software.

### 2.4. Quantification of intracellular cytokine production and activation marker

For determination of mediator production and activation marker by PBMCs, cells were activated and treated with psychedelic compounds or CsA for 48 h. PBMCs were then re-stimulated using phorbol-12-myristate-13-acetate (PMA; 50 ng/ml) and ionomycin (500 ng/ml; both from Sigma) and incubated with BD Golgi Plug™ (BD Biosciences, Allschwil, Switzerland) for 4 h. After washing, cells were fixed with 4% paraformaldehyde (PFA; Morphisto, Offenbach am Main, Germany), and staining of intracellular cytokines and markers was carried out using fluorochrome-conjugated anti-human mAbs (BioLegend, Amsterdam, Netherland, and Beckman Coulter, Zurich, Switzerland). The levels of cytokine production and activation marker expression were determined using flow cytometric analysis of three independent experiments.

### 2.5. Evaluation of NF-κB expression

The human cell line tagged with an enhanced green fluorescent protein (eGFP) THP-1 NF-κB-eGFP was purchased from Merck (Zug, Switzerland) and is derived from a single-cell clone of THP-1 cells stably transfected with an NF-κB-eGFP reporter construct (26). The parental THP-1 cell line was derived from the peripheral blood of a 1-year-old male suffering from acute monocytic leukemia (27). The cells were cultured in Roswell Park Memorial Institute (RPMI) 1,640 medium (Invitrogen) supplemented with 10% heat-inactivated fetal calf serum (PAA), 2 mM L-glutamine, 100 U/ml penicillin, and 100 U/ml streptomycin (Invitrogen) at 37°C in a humidified incubator with a 5% CO_2_/95% air atmosphere. The cells were stimulated with lipopolysaccharide (LPS: 100 ng/ml, Sigma). Incubation was carried out for 24 h in the presence of medium alone, control substances, and psychoactive compounds. The levels of NF-κB expression reflected by eGFP were determined using flow cytometric analysis of four independent experiments.

### 2.6. Statistical analysis

Data were statistically analyzed using GraphPad Prism (Version 9.3.1, San Diego, CA, USA). The effects of each drug on proliferation rate, cytokine release, and NF-κB induction were evaluated using one-way ANOVA (Brown-Forsythe and Welch ANOVA tests) followed by Dunnett’s T3 multiple comparisons test to compare drug effects to stimulated control cells. *P < 0.05 was used as the minimum criterion for statistical significance for all experiments.

## 3. Results

### 3.1. Psychedelics do not affect primary human T cell proliferation

**FIGURE 1:**
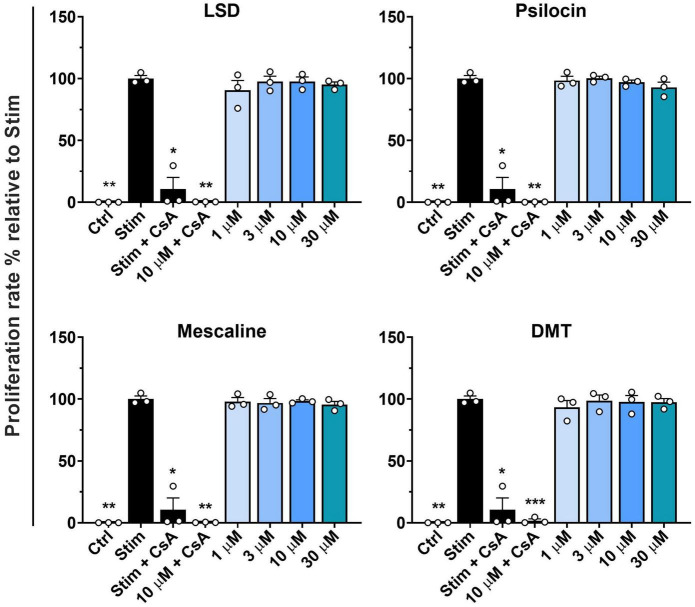
Influence of classic psychedelics on the proliferation of primary human T lymphocytes. PBMCs were stimulated with anti-human CD3 and anti-human CD28 mAb (Stim) and incubated in the presence of medium alone (Neg Ctrl), different control substances, and psychedelic compounds for 72 h. Cyclosporine A (CsA) served as a control substance for proliferation inhibition. Data are the mean ± SD from three independent experiments. Data were analyzed by one-way ANOVA followed by Dunnett’s T3 multiple comparison test (***P < 0.001, **P < 0.01, *P < 0.05 when compared to stimulated cells without drug treatment).

First, we assessed whether the classic psychedelics LSD, psilocin, mescaline, and DMT inhibit the proliferation of stimulated primary human T cells. The used concentration range of 1–30 μM represents suprapharmacological concentrations of LSD, psilocin, and DMT, when compared to maximal plasma concentrations measured in clinical studies (200 μg LSD oral: 12.1 ± 1.2 nM, 30 mg psilocin oral: 97.9 ± 26.4 nM, 1 mg/min DMT i.v. for 90 min: 236.4 ± 67.5 nM) (28–30). Mescaline plasma concentrations after a 200 mg oral dose were in the range of 4 μM (31). As shown in Figures 1A–D, none of the tested psychedelics affected the proliferation rate at concentrations of up to 30 μM. In contrast, the positive control CsA strongly inhibited T cell proliferation. However, the addition of 10 μM of either psychedelic compound to CsA accentuated proliferation inhibition.

### 3.2. Activation and mediator release of primary human CD8^+^ T cells by psychedelics

**FIGURE 2:**
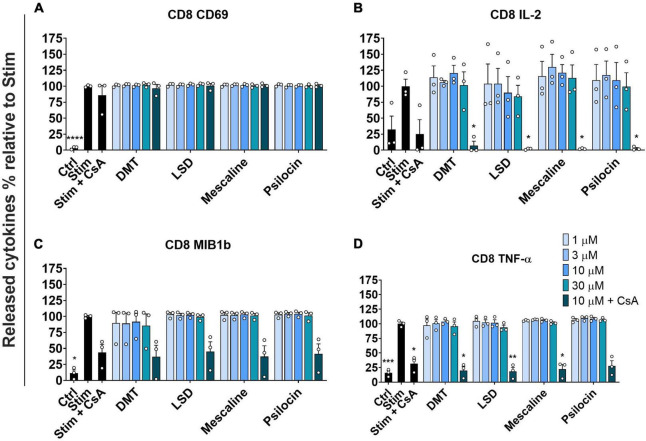
Influence of classic psychedelics on **(A)** the activation (CD69) and function/mediator release in form of **(B)** IL-2 release, **(C)** CC-chemokine ligand 4 (MIB1b) release, and **(D)** TNF-α release of primary human CD8^+^ T cells. PBMCs were stimulated with anti-human CD3 and anti-human CD28 mAb (Stim) and incubated in the presence of medium alone (Neg Ctrl), different control substances, and psychedelic compounds for 48 h. PBMCs were re-stimulated with PMA and ionomycin for 4 h. Cyclosporine A (CsA) served as a control substance for cytokine release inhibition. Data are the mean ± SEM from three independent experiments. Data were analyzed by one-way ANOVA followed by Dunnett’s T3 multiple comparison test (****P < 0.0001, ***P < 0.001, **P < 0.01, *P < 0.05 when compared to stimulated cells without drug treatment).

We assessed the activation of stimulated primary human CD8^+^ T cells by determining CD69 expression as an early marker of lymphocyte activation (32). As depicted in Figure 2A, none of the tested psychedelics reduced CD69 expression in stimulated CD8^+^ T cells. However, CsA did not significantly reduce CD69 expression in stimulated CD8^+^ T cells. Next, we determined the released interleukin-2 (IL-2), which acts as a growth regulator of T cells *in vitro* and supports the proliferative expansion of cytotoxic T lymphocytes (33). Figure 2B shows that none of the tested psychedelics significantly affected IL-2 release in stimulated CD8^+^ T cells. In contrast, CsA significantly reduced IL-2 release, which was even more pronounced by the addition of 10 μM of either psychedelic compound.

Moreover, we assessed the potential effect of psychedelics on CC-chemokine ligand 4 (MIB1b) release, which has been shown to be up-regulated in patients with type 2 diabetes mellitus and clinical atherosclerosis cardiovascular diseases (34). As shown in Figure 2C, none of the tested psychedelics significantly affected the release of CC-chemokine ligand 4 in stimulated CD8^+^ T cells. However, the positive control CsA significantly reduced the release of CC-chemokine ligand 4, which was not further accentuated by the addition of either psychedelic.

To complement the assessment of stimulated primary human CD8^+^ T cell activation, we investigated the release of TNF-α. TNF-α release is regulated by various functions, including cell growth modulation, tumorigenesis, inflammation, and autoimmunity (35, 36). Figure 2D shows that none of the tested psychedelics had a significant effect on TNF-α release. In contrast, CsA significantly reduced the release of TNF-α, which was not further accentuated by the addition of either psychedelic.

### 3.3. CD69 activation and mediator release of primary human CD4^+^ T cells by psychedelics

**FIGURE 3:**
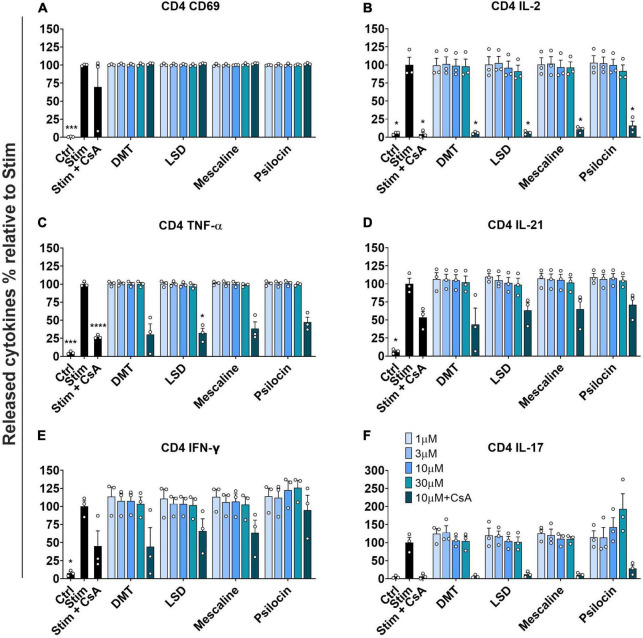
Influence of classic psychedelics on **(A)** the activation (CD69) and function/mediator release in form of **(B)** IL-2 release, **(C)** TNF-α release, **(D)** IL-21 release, **(E)** IFN-γ release, and **(F)** IL-17 release of primary human CD4^+^ T cells. PBMCs were stimulated with anti-human CD3 and anti-human CD28 mAb (Stim) and incubated in the presence of medium alone (Neg Ctrl) and psychedelic compounds for 48 h. PBMCs were re-stimulated with PMA and ionomycin for 4 h. Cyclosporine A (CsA) served as a control substance for cytokine release inhibition. Data are the mean ± SEM from three independent experiments. Data were analyzed by one-way ANOVA followed by Dunnett’s T3 multiple comparison test (****P < 0.0001, ***P < 0.001, *P < 0.05 when compared to stimulated cells without drug treatment).

Next, we investigated the activation of stimulated primary human CD4^+^ T cells by determining the CD69 expression and the release of various mediators. Similar to the results obtained in stimulated CD8^+^ T cells, none of the tested psychedelics reduced CD69 expression in stimulated CD4^+^ T cells (Figure 3A).

Moreover, none of the tested psychedelics significantly affected IL-2 release in stimulated CD4^+^ T cells. In contrast, the included positive control CsA significantly reduced IL-2 release, which was not further accentuated by the addition of either psychedelic compound (Figure 3B).

Figure 3C shows that none of the tested psychedelics had a significant effect on TNF-α release up to 10 μM. However, at a concentration of 30 μM, each tested compound reduced TNF-α release below 50%, which was in the same range as the combination of CsA and 10 μM of the respective compounds.

Moreover, we assessed the potential effect of psychedelics on IL-21 release, which is needed to propel central and effector memory CD8 T cell differentiation (37). None of the tested psychedelics significantly affected IL-21 release in stimulated CD4^+^ T cells. In contrast, the included positive control CsA reduced IL-21 release to around 50%, which was not further accentuated by the addition of either psychedelic compound (Figure 3D).

Next, we investigated the effect of psychedelics on the release of interferon-gamma (IFN-γ), which serves as a crucial inducer of immune effector mechanisms between innate immune cells and effector memory T cells (38).

Figure 3E shows that none of the tested psychedelics significantly reduced IFN-γ release in stimulated CD4^+^ T cells. However, CsA significantly reduced IFN-γ release, which was not further accentuated by the addition of either psychedelic.

Moreover, we assessed the potential effect of psychedelics on IL-17 release, which is associated with various inflammatory autoimmune diseases (39).

As shown in Figure 3F, none of the tested psychedelics significantly affected IL-17 release in stimulated CD4^+^ T cells. In contrast, CsA significantly reduced the release of IL-17, which was not further accentuated by the addition of either psychedelic.

### 3.4. Effect of psychedelics on LPS-triggered NF-κB induction of THP1-NF-κB reporter cells

**FIGURE 4:**
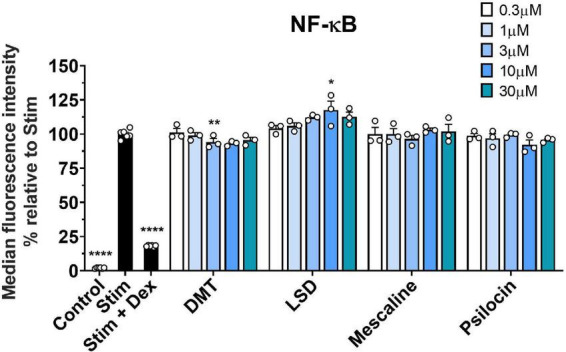
Effect of classic psychedelics on the LPS-triggered GFP-NF-κB induction of THP1 NF-κB reporter cells. THP-1 NF-κB-eGFP cells were stimulated with LPS (Stim) and incubated in the presence of medium alone (Ctrl) and classic psychedelics for 24 h. Dexamethasone (Dex) served as a control substance for NF-κB inhibition. Data represent the mean ± SD from four independent experiments. Data were analyzed by one-way ANOVA followed by Dunnett’s T3 multiple comparison test (****P < 0.0001, **P < 0.01, *P < 0.05 when compared to stimulated cells without drug treatment).

Since we did not observe any relevant immune-modulatory effects of classic psychedelics in the investigated T cell lines, we included the human monocytic THP-1 cell line to monitor the NF-κB signal transduction pathway. Monocytes contribute substantially to pro-inflammatory immune responses in humans, and the transcription factor NF-κB represents a central mediator of inflammation (40, 41). Thus, we assessed the effect of classic psychedelics on the LPS-triggered GFP-NF-κB induction. As shown in Figure 4, none of the tested psychedelics reduced the NF-κB signal compared to the control incubations. However, dexamethasone, which was included as a positive control, significantly reduced the NF-κB signal. Hence, we do not expect LSD, psilocin, DMT, or mescaline to affect the monocytic immune reaction *via* the NF-κB signal transduction pathway in humans.

## 4. Discussion

The results of the current study indicate that the classic psychedelics LSD, psilocin, DMT, and mescaline do not directly modulate the proliferation or stimulated cytokine release of human T lymphocyte subpopulations. Moreover, classic psychedelics have no effect on the stimulated NF-κB signal in monocytes. Since classic psychedelics are utilized in assisted psychotherapy in patients with life-threatening diseases such as end-stage cancer (4, 42), suppressing effects on lymphocytes or monocytes are unwanted. Immune-suppressive effects of psychedelics would hamper the future treatment of patients with already impaired immune systems. However, worsened immune system function after psychedelic-assisted psychotherapy has not yet been observed. In contrast, the safety of psychedelic treatments has been reported in several studies (24, 43–45), which is in line with the results of the current study.

Nevertheless, studies in PBMCs and animal models have reported potent anti-inflammatory effects due to 5-HT_2*A*_ or 5-HT_1*A*_ receptor activation (16, 20, 21, 46, 47). A study investigating the significance of 5-HT in inflammation showed that 5-HT and DOI inhibited TNF-α production in LPS-stimulated PBMCs. Moreover, the inhibitory effect of 5-HT and DOI on TNF-α production was associated with the activation of the 5-HT_2*A*_ receptor (47). However, all 5-HT and DOI concentrations used in this study (up to 100 μM) that inhibited TNF-α production were much higher than any plasma concentrations detected in humans and could therefore be considered not clinically relevant. In addition, Cloëz-Tayarani and colleagues (47) did not observe any relevant effect of 5-HT or DOI on the production of various interleukins, which is in line with our observations. Yu and colleagues (16) showed that DOI reduces the effects of externally added TNF-α in mouse primary aortic smooth muscle cells, inhibiting various TNF-α-mediated pro-inflammatory markers. Since the effects of DOI on externally added TNF-α were investigated, these findings do not contradict the results of the current study. We analyzed the effect of classic psychedelics on the release of TNF-α from primary CD4^+^ and CD8^+^ T cells, which was not affected. However, subsequent effects of TNF-α-mediated pro-inflammatory markers were not part of the current investigation. Studies investigating the association of 5-HT_1*A*_ receptor activation and immune modulation showed that the potent and selective 5-HT_1*A*_ receptor agonist DPAT induces lymphocyte proliferation probably by increased translocation of NF-κB into the nucleus (20, 21). However, none of these studies observed immunomodulatory effects of psychedelics, although several psychedelics potently activate the 5-HT_1*A*_ receptor (48). We, therefore, assume that the in the current study tested psychedelics are, in contrast to DPAT, not potent or selective enough 5-HT_1*A*_ receptor agonists to induce immune modulation.

In the current study, drug effects were assessed after 24–72 h treatment, whereas a *in vivo* study in healthy volunteers reported peak effects of CD3 and CD4 decrease after 2 h (18). In contrast to the *in vivo* situation, lymphocytes isolated *ex vivo* do not show stable and meaningful cytokine production after such short stimulation periods and therefore cannot be reliably analyzed earlier at the secretion level, since at least 24–48 h are required. Moreover, to assess the cell proliferation parameter, an experimental period of 48–72 h is required to observe meaningful division of the cells *in vitro*. The above-mentioned *in vivo* study (18) analyzed the percentage of certain subpopulations in the blood as well as neuroendocrine factors, whereas in the current study the cytokine secretion by lymphocytes was assessed.

The same study in healthy volunteers showed that both DMT and d-amphetamine significantly decreased peak CD3 and CD4 levels shortly after treatment. Due to the remarkable similarity between the effects of DMT and d-amphetamine, whereof d-amphetamine is no 5-HT_2*A*_ receptor agonist, the authors concluded that the observed effects were caused by an indirect mechanism rather than specific drug–target interactions with immune cells (18). Hence, they showed that increased cortisol levels due to DMT and d-amphetamine treatment caused decreased peak CD3 and CD4 levels. These results are in line with the observations of the current study, showing that classic psychedelics do not directly affect primary T lymphocytes and monocytes. The mixed stimulant-psychedelic substance 3,4-methylenedioxymethamphetamine (MDMA), which is also used in substance-assisted therapy, similarly acts as a cortisol releaser (49) and also decreases CD4 T-helper cells and the functional responsiveness of lymphocytes to mitogenic stimulation. In addition, MDMA increased natural killer cells and some anti-inflammatory cytokines (transforming growth factor-β and IL-10) but reduced anti-inflammatory IL-2 (50). The activation of the sympathetic nervous system and cortisol release have a known modulatory effect on lymphocytes (51). Similar to the above-mentioned results of Dos Santos and colleagues (18), psilocybin and LSD administrations have been shown to acutely increase cortisol levels in humans (52, 53). It has been suggested that psychoactive substances stimulate stress responses through the 5-HT_2*A*_ receptor and the associated cortisol release (54). Taken together, this might explain the immune-modulatory effects of classic psychedelics observed in humans or animal models that were attenuated by 5-HT_2*A*_ receptor inhibition (18, 52, 55, 56). Hence, classic psychedelics, as well as other cortisol release–stimulating compounds such as d-amphetamine, seem to have indirect anti-inflammatory effects. However, these effects are not elicited by direct stimulation of T lymphocytes or monocytes.

## 5. Conclusion

The classic psychedelics LSD, psilocin, DMT, and mescaline do not directly stimulate the proliferation or cytokine secretion of primary human T lymphocytes or monocytes. However, classic psychedelics, as well as other psychoactive substances, may induce cortisol release potentially through 5-HT_2*A*_ receptor activation, leading to anti-inflammatory effects in humans and animal models. These findings are of significance for the future safe use of classic psychedelics in assisted psychotherapy in patients with life-threatening diseases.

## Data availability statement

The raw data supporting the conclusions of this article will be made available by the authors upon request, without undue reservation.

## Ethics statement

Ethical review and approval was not required for the study on human participants in accordance with the local legislation and institutional requirements. The patients/participants provided their written informed consent to participate in this study.

## Author contributions

AA performed all experiments and analyzed the data with assistance of CG. DR wrote and revised the manuscript with significant inputs from ML and CG. All authors contributed to the article and approved the submitted version.
